# Supplemental Review, or Quarterly Periscope of Practical Medicine and Surgery

**Published:** 1822-06-01

**Authors:** 


### Supplemental Review

AND

### QUARTERLY PERISCOPE

OF

### PRACTICAL MEDICINE, SURGERY, &c. &c.

### *WITH COMMENTARIES.*

*Paucis libris immorari et innutriri oportet, si velis aliquid trahere, quod in animo fideliter hæreat.* SENECA.

Duo vitia vitanda sunt in cognitionis et scientiæ studio. ***** Alterum est vitium, quod quidam nimis magnam operam conferunt in res obscuras atque difficiles, easdemque non necessarias. CICERO.

IN opening our periscopic budget for this quarter, we shall quote a passage from the second volume of LACON, recently published. It is not inapplicable to our present purpose. "An æra," says the author, "is fast approaching, when no writer will be read by the "great majority, save and except those, who can effect for bales of "manuscript, what the hydrostatic screw performs for bales of cotton "—by condensing that matter into a period, which before occupied "a page."* The able author of Lacon goes on to state that, "he "has attempted to make an intelligible book, capable of doing some "good to that valuable class of the community who have *other* things "to do, as well as to read; and who, when they snatch a few hours "from their occupations to devote to literary pursuits, must necessarily prefer that book which affords them the most knowledge, and "takes from them the least time."† If these observations are applicable to the community at large, they are peculiarly so to the class of medical society, whose daily avocations are of the most harrassing nature, and whose "literary pursuits" are not merely recreations that may or may not be indulged in, but essential requisites for the invigoration of their intellects and the performance of their duties. The farther we proceed, the more convinced we become of the great importance of this feature, (Quarterly Periscope), in a periodical Journal of Medical Science. But to make it practically useful, we find it necessary to restrict it within a certain range. Were we to attempt a general view of *all* that is going on in the medical world, we should

* Lacon; or Many Things in Few Words, Vol. II. p. 91.

† Ibidem, p. 91.

then, do little more than tantalize our readers with a steril catalogue of "insubstantial pageants," that would scarcely leave a single imprint of practical precept on the mind. Of those subjects then, which we notice, we shall endeavour to convey a clear idea to our readers —and more than this, we have neither inclination to promise, nor time to perform.

It has been but too remarkable, of late years, that the taste of the present times does not lead many practitioners to contribute to the monthly medical journals, as was formerly the case. Into the causes of this change, we do not think it necessary to inquire; but we regret the fact, and we should be glad to see things come back once more into the good old channel. When a man contributes a paper or an observation to a periodical journal, his wish must naturally be, that it may obtain the greatest possible publicity. As a stimulus to the said contributions, we promise to notice in our Periscope, all the more interesting papers in the original departments of our cotemporaries; and we need hardly observe that, in this way, they will not want for publicity. Neither need the contributors be apprehensive of that personal and malignant criticism, which has too often disgraced the periodical press in all countries. Candid commentary will be occasionally indulged in—waspish censure never.

---

1. *Tic Douloureux.** A poor woman experienced the miseries of the damned, (if indeed the inhabitants of the nether world are subject to nervous disorders) for 20 years—that is, from the age of 50 to that of 70, with tic douloureux. She was blistered, purged, salivated, leeched, carved from ear to ear, where there were nerves, and we had almost said, where there were no nerves; poisoned with arsenic, greased with belladonna, conium, tar-varnish and soap-liniment—narcotized with opium—carbonized with iron-rust—edentified by the tooth-drawer—and all with scarcely a mitigation of her dreadful sufferings! At length, upon minute investigation, it appeared, that the alvine evacuations had long been in an unhealthy state—"sometimes scanty, dark-coloured, and scybalous—at others, profuse, acrid, and mottled—her appetite either voracious or the reverse—her tongue furred—pulse feeble and quick—skin alternately affected with chills and hot flushes—her nights tormenting, and her days but little better." She was first put upon the constant use of the mildest aperients until the evacuations were improved, when a strong decoction of cinchona infused upon quassia, was regularly taken in as full a dose as the stomach would admit, exhibiting, at the same time, a grain and a half of pure opium twice a day, constipation being guarded against by sulphate of magnesia. In this way, some ground was gained; but still the patient's sufferings were great. It was, therefore, determined again to have recourse to arsenic, be-

* Mr. George Nesse Hill. *Ed. Journal, No.* 71.

ginning with doses of three drops of the arsenical solution, and gradually increasing it to twelve drops thrice a day, omitting the opium, except when severe paroxysms occurred. When arrived at nine drops, she was better, and expressed confidence in the plan. At the end of a month, being free from pain, and the system being evidently under the influence of arsenic, its further use was discontinued *gradatim.* She continued well for several weeks, until the bowels again became irregular. Then a ptyalismic soreness attacked the mouth. Twitchings soon followed—and lastly, a bearable degree of the old evil. But, on regulating the bowels and returning to the solution, all was speedily corrected, and the medicine a second time left off. Three months afterwards, the bowels were much affected with diarrhœa; but no shocks of the old misery returned. She is now in good health and spirits. Long may she remain so, pray we! But we have seen and known several instances of this deceitful remission in tic douloureux, and, therefore, we are doubtful of cures till many months have established their solidity.

We have long observed that, although *violent pain* of a part, generally manifests itself in that part; yet, *protracted irritation* of a nervous structure, more usually shews itself at a distance—and that, sometimes, with excruciating agony. Tic douloureux exemplifies this remark. How rarely is the cause of the pain seated in the nerve which expresses it! The splanchnic nerves being devoid of *common sensibility*, seem, very generally, to manifest their irritation through the medium of one of the common cranial or spinal nerves. Many of our readers may remember some notice which we published, in the 7th No. of our last, or Quarterly Series, (January 1820,) of a gentleman afflicted with dreadful tic douloureux in the ancle. We lost sight of him for a considerable time, after prescribing gentle and mild aperients, with a slight mercurial alterative, (four grains of the blue pill and four of rhubarb,) twice a week. We directed him, after persevering in this plan for a good while, to take five grains of the oxyde of bismuth three times a day, for two, three, or four weeks. We met him in the street about a fortnight ago, in comparatively good health. His old enemy now troubled him but seldom, and then with greatly mitigated violence. He still persevered with gentle aperients, and occasionally the blue pill.

In a conversation which we lately had with Sir Henry Halford, on the subject of this complaint, that experienced physician mentioned several remarkable cases, many of them in high life, where it ultimately turned out, that some *bone* was diseased and kept up this sympathetic irritation in the fifth pair of nerves. This hint ought to be borne in mind, and may induce medical men to make particular enquiries of their patients whether they have had falls, or other accidents that might be likely to injure some part of the bony structure, or render it carious. One of the cases mentioned by Sir Henry Halford, was that of an officer who had lost a limb, and afterwards became affected with neuralgia facialis, which nothing could make an impression on. At length, a piece of bone exfoliated from the stump, and the neuralgia disappeared.

2. *Cephalitis.** Dr. Blicke, a zealous practitioner, has related an interesting case of inflammation of the brain, with disorganization of the corpora striata, and lastly, effusion of blood. The patient was an unmarried lady, 33 years of age, who had laboured for some years, under, what were termed, dyspeptic and bilious complaints; but, without any very manifest advantage from medicine. On the 21st November, Dr. Blicke was called to the patient, and found her complaining of great head-ache, attended with a train of dyspeptic symptoms. The pulse was 120, small, contracted, and weak. Venesection was proposed, but not acceded to. Regulated diet, alteratives, and enemata were prescribed: and under this treatment, the patient apparently got rid, not only of the head-ache, but of all the dyspeptic symptoms. On the 8th December, she complained of sore mouth—a crop of aphthæ appeared—and the bowels evinced an affection of the same kind, pervading their internal surface. These symptoms yielded to bark, cordial diet, and warm laxatives. At this time, however, Dr. Blicke perceived a quickness in her answers, attended with unusual irritability. Notwithstanding the use of aperient medicines, local evacuations, saline mixtures with antimony, and gentle sedatives, this irritability increased, the celerity of the pulse was on the advance—and her nights were restless. Dr. W. Philip met Dr. Blicke in consultation; at which time the patient was incoherent—the tongue red and glairy, with fur towards the centre—pulse 130 and small—slight pain and weight in the head—stools dark and offensive. Leeches were again applied to the forehead—saline mixture with hyoscyamus internally. She died that night, being about the 20th December.

*Dissection.* A small clot of blood was found between the dura mater and tunica arachnoidea, over the right and posterior lobe of the cerebrum—about an ounce of apparently arterial blood rested on the tentorium. The effusion could not be traced to any evident rupture of a blood-vessel. There were a few opake spots in the arachnoid—and on the top of the left hemisphere there was a piece of coagulable lymph, the size of a shilling. The sectio ovalis exhibited those numerous red points usually seen in inflammation of the brain. The lateral ventricles were somewhat distended with a reddish turbid serum. The right corpus striatum was harder than usual, "and had evidently a scrophulous appearance, with a cheese-like feel." Here a small excrescence, about the size of a very large pea, exhibited an apparent ulceration in its centre. The left corpus striatum was softer than usual, "and on cutting into it, an abscess was perceived, containing a small quantity of pus." The villous coat of the stomach, duodenum, and jejunum, was highly vascular. The pylorus was considerably constricted and indurated.

This case shews the obscurity, treachery, and fatality, of affections of the brain. The subject will undergo ample discussion in our next number.

---

* Med. Repository, March, 1822.

3. *Fracture of the Patella.** We were rather surprized to find Mr. Fielding remark that, *transverse* fractures of the patella are of rare occurrence, when compared with *longitudinal* fractures. We believe that, in all cases where the fracture occurs from the action of muscles, it will be found to be transverse, while in accidents *ab externo*, it may be transverse or longitudinal according to circumstances. Be this as it may, our author advocates the practice of effecting an *osseous* union, if possible. Dupuytren advocated the same practice some years ago, and thought it possible to effect the bony union by elevating the trunk of the body, and then raising the heel of the injured limb to a certain angle with the pelvis. Mr. Wilson, also, in his late work, observes that, in the collection of Dr. W. Hunter, there was one well-marked instance of bony union in transverse fracture of the patella, and that he has seen other instances in the dead body of its having occurred. Professor Sheldon has recommended the patient to be placed on either side, with the hip bent, and the knee a little bent also—the degree of inflexion of the hip to be equal to the bringing down the superior portion of the patella to unite with the inferior —a mode of practice which, he says, has produced a perfect bony union of the fractured patella. As the case on which this little pamphlet is founded, will occupy but a small space in our pages, we shall give it in the author's own words.

*Case.* "Mrs. H. æt. 35, a tall well proportioned woman, on the 13th *Nov.* 1820, lifting a heavy basket while in the erect position— her left knee suddenly gave way and she fell to the ground. Upon visiting her immediately afterwards, it was evident that the left Patella was fractured transversely near its middle. The ends of the fractured bone were above an inch asunder. She was put to bed upon a hair mattress—the trunk and shoulders were well elevated. When the affected limb was placed horizontally, the ends of the fractured Patella remained about the same distance from each other as before.† The heel was now gradually elevated by my son, who was my assistant at the time, until the fractured portions were brought into close apposition. In this situation they were secured by straps of adhesive plaster put three parts round the limb, above and below the Patella, by bandage passed *moderately tight* above and below the knee in the ordinary way. A splint about twelve inches long was placed under the ham and slightly secured by bandage. The elevated position of the heel was strictly preserved—the whole limb was thus placed upon an inclined plane from the heel to the hip—the heel being the highest. The Patella being left nearly uncovered, it was easy now to feel that the fractured bone was in exact contact. The knee was kept moist by a cold lotion. No great pain and very

---

* Case of Transverse Fracture of the Patella, &c. By Mr. George Fielding. Octavo, sewed, 1822.

† We tried the position recommended by Professor Sheldon, but did not succeed in bringing the fractured bone into contact by that plan.

little swelling ensued. The chief inconvenience which the patient experienced was from the stretching of the flexor muscles, she was however perfectly manageable and preserved her position with the utmost care, from an apprehension that eventual lameness might ensue from the injury, if by any unguarded motion the fractured bone should be displaced, and the cure rendered imperfect.

"At the end of a fortnight the bandages were replaced. It was then clear, that the fractured ends of the bone were in contact, from the tumefaction upon the Patella, and from the exact situation of the fracture being no longer distinguishable.

"On removing the bandages at the end of a month, the bone was found to be firmly united, without any intervening ligament whatever.—Only a small hard line a little elevated was discoverable in the course of the fracture. The heel was now brought down to a horizontal position—the shoulders were lowered—the splint and a bandage loosely applied were kept on at the request of the patient a week longer—and gentle passive motion employed every day:—This woman left her room before the end of the sixth week. Diligent friction and frequent passive motion of the joint were necessary for some time. The flexibility of the joint and the power of the limb were gradually restored, and the patient now enjoys the use of the limb in every respect as perfectly as before the accident." 12.

We think Mr. Fielding's pamphlet would have been better as an article in some periodical journal, than as a separate publication. But, as the main facts, and the case itself, will now receive a wide enough circulation, Mr. Fielding's object will be completely attained.

---

4. *Dislocated Thigh Bone.** Mr. Cornish of Falmouth, has related a case of this kind, which may induce hopes of reduction at a later period than is usually supposed within the reach of cure. The patient was a seaman, first treated at St. Thomas's Hospital for fracture of the neck of the thigh bone, and discharged with the assurance that the limb would be useless for life. He was afterwards admitted into Guy's Hospital, when Sir Astley Cooper pronounced it dislocation, and tried all means of reduction without effect. He was therefore dismissed as an incurable cripple. About twelve months after the accident, viz. in 1813, he presented himself before Mr. Cornish, at the Falmouth Dispensary, on crutches, and gave the foregoing account.

"On examining him," says Mr. Cornish, "I found the injured limb about two inches and a half shorter than the other, entirely useless, producing great pain on putting it to the ground, and the knee and foot turned inwards. There was considerable distortion about the joint, and the head of the bone appeared to have formed a socket

---

* Med. Repository, March, 1822.

for itself on the dorsum ilii. In short, he had every diagnostic symptom of the dislocation upwards, which Sir A. Cooper has so accurately marked in his valuable essay on this subject. In consequence of the duration of the accident, and the failure of the attempts at reduction under the skilful management of Sir A. Cooper, his case was considered irremediable, and nothing was done for him. In March, 1818, I met the man walking without the least degree of lameness, carrying a heavy basket on each arm. On satisfying myself that he was the patient I had examined at the Dispensary, and on inquiring into the cause of his cure, he informed me, that, in the summer of 1817, five years after the accident, whilst on a passage from Falmouth to Plymouth in a little coasting vessel, the ship made a lurch, which knocked him down. At the moment he fell, he heard a loud crack in his hip; and, from that time, he put aside his crutches, and perfectly recovered the use of his limb. The man is now doing duty as an able seamen on board a ship which trades from this port to London." 201.

Mr. Cornish saw Sir Astley Cooper reduce a dislocation of the hip-joint of six months standing, which he heard Sir A. say was the most protracted case in which he had had success.

---

5. *Traumatic Tetanus cured.** Messrs. Mercier and Parant, of Quebec, have recently encountered a case of this kind, in a man, 50 years of age, who, some days after being wounded in the foot by a large nail, became affected with opisthotonos. Our authors bled *ad deliquium*, and exhibited strong mercurial and other purgatives. The venesection was several times repeated, and the warm bath was employed. The patient recovered. It may be proper to state that the wound was re-opened by our authors when called in, and that burning spirits were applied to it to keep it open afterwards. Great difficulty was experienced in producing purgation.

---

6. *Nervous Paralysis.*† We are very far from agreeing with M. Fouquier in the propriety of the name given to the disease in question, and our reasons will be seen as we proceed in the details of the case, which is an important one.

" A young woman, 19 years of age, very stout, of full sanguineous habit, with regular but *scanty* menstruation, experienced, on the 27th November, a loss of strength in the lower extremities, shortly after which the upper limbs also lost their power of voluntary motion. At the same time she felt vertigo, and noise in her ears; the

---

* Med. and Phys Journal, Dec. 1821.

† Paralysie Nerveuse. Par M. Fouquier.—*Annuaire Med. Chir.*

intellect, however, remaining unaffected. Leeches were applied to the anus and to the neck. On the 1st December she entered La Charité; and on the 2d, presented the following phenomena:—total loss of voluntary motion in the lower extremities, and partial loss in the upper:—sensibility unaffected in either. The patient was perfectly collected, and rendered an exact account of herself. She complained of dimness of vision, and tinnitus aurium. Her countenance was flushed, and a little tumid—the pupils dilated—the left angle of the mouth drawn a little backwards and upwards—the right in a contrary direction—her tongue inclined to the left when put out. The pulse was full and strong, but not quickened in number. All the signs of plethora being united in this girl, M. Fouquier ordered the jugular vein to be opened, but the quantity of blood drawn is not specified. Lemonade and cream of tartar for drink. *3d. Dec.* Countenance still flushed—the distortion about the mouth less striking—considerably more power in the upper extremities—the lower remained motionless—tinnitus aurium. Another bleeding from the arm; but the quantity and effect on the system not stated. 4th. Less power in the arms than yesterday—breathing difficult—pulse accelerated. Some evacuations provoked by an emetico-cathartic draught—a blister to the nucha—patient died in the evening.

" *Dissection.* The exterior vessels of the brain, and those of the plexus choroides, were full of blood—and blood appeared in numerous points of the brain at each cut of the scalpel. The substance of the brain itself was firm. No extravasation in the ventricles or in any part of the head or spinal canal." 376.

In the first number of this series, page 10, we made some observations on this case, and shewed that there were unequivocal marks of general compression of the brain, which often led to the most fatal species of apoplexy and paralysis, without any extravasation of blood beyond the parietes of the vessels. To M. Fouquier's question then—"*La plenitude des vaisseaux sanguins du cerveau est-elle la seule cause de cette paralysie génèrale et de la mort?*" We answer yes; and we consider it by far the best and the safest conclusion to draw, whether we look to pathology or practice. The inert treatment in this case is a pretty significant commentary on the doctrine of "*nervous* apoplexy and paralysis," so commonly in the mouths of continental physicians.

---

7. *Puncturing Anasarcous Limbs.** We confess that we were not a little amused by this elaborate effusion of "a member of the Royal College of Surgeons," who, with some naiveté, believes that the measure here recommended will "prove itself to be of far greater utility in evacuating the fluid diffused through the cellular tissue of the body, *than any eulogium* he can bestow in favour of its restora-

---

* Med. and Phys. Journal, No. 279.

tion." If indeed it have not infinitely more effect than a *eulogium* in removing dropsical fluids, we fear its restoration is hopeless! The *facts* detailed in support of the measure are in the form of a *supposition.* "Suppose I were called to A. B, &c" we shall not therefore analyse this supposed case of "anasarca of the lower half of the body, in consequence of *palpitation* of the heart," because we believe that no such case ever existed. There is a passage at page 363, which augurs but little of the great success attending this puncturing of anasarcous legs. "It has *often*," says he, "struck me with utter surprise at finding patients dead a few days after this operation was resorted to—patients who had not the appearance of so immediately dying, at the time it was performed." This led our author "into many *endless* difficulties," which, however, in the next line proved *not* to be endless, for, like a ship coming to an anchor, he dexterously "extricated himself by *bringing up* to the following conclusions." These conclusions were, that the said sudden deaths "were attributable to the removal of the accumulation of the lymph, which, in the relaxed state of the system at this time, supported the circulation by means of its pressure, &c." For our own parts we would humbly hint that this honest gentleman need not accuse himself of the death of these patients for not having applied a bandage after the scarifications. The causes that produced the œdema of the lower extremities were far more likely to induce sudden death than the scarifications. After puncturing and bandaging, he knows of no medicine better to make a trial of "than fl. ʒj. of the tincture of digitalis, in fl. ʒiij. of the spiritus ætheris nitrici." If our author means this for one dose, there is *rather* too much of the digitalis. If he means the usual dose of digitalis to be given from the above mixture, then the quantity of spir. ætheris nitrici in each dose will avail nothing. We are sorry to see papers of the above description so carelessly sent forth to the world. Such papers deserve gentle censure.

8. *Phthisis.** Dr. Carson's ideas respecting the circulation of the blood, and the elasticity, or, as he terms it, *resiliency* of the lungs, as an agent in that mysterious process, have long been before the public. He has lately *proposed* the application of his theory to practice. Considering, and doubtless with justice, that the lungs are at all times in a forced state of dilatation, (we mean while capable of respiration,) he attributes to this circumstance the difficulty of healing any lesion in them. And as experiments on animals, and accidents in man, have proved that air may be admitted into one side of the chest without much danger, Dr. Carson proposes that the operation be performed in cases of phthisis, in order that the lung of the side diseased may be rendered quiescent, and thus the abscess have time to heal.

* Essays, Physiological and Practical. By Dr. Carson, of Liverpool.

We are sorry that we cannot go along with Dr. Carson in this proposal, nor at all agree with him in the propriety of it. In the first place, we are far from believing that confirmed phthisis consists merely in a common abscess of the lungs. The tubercular state which so long precedes the discharge of purulent matter by expectoration —the repeated inflammatory processes which have occurred—the adhesions and other lesions which take place in the course of the disease, all combine to bring the respiratory organ into a state which could not be bettered, and would almost to a certainty be rendered worse by the operation of admitting air into the thoracic cavity.

In the second place, we believe that puncture of the pleura would very seldom lead to collapse of the lung in confirmed or purulent phthisis, on account of the adhesions which are so very generally found in that stage of the disease. In the third place, how could we be certain of the side where the disease is situated, even with the aid of percussion and auscultation? How seldom, indeed, do we find the disease confined to one lung in phthisis. And after all, even if collapse of the lung did take place, would that enable us to heal a scrophulous abscess in the chest, when we find so much difficulty in healing a similar one on any external part, however easy and quiet we keep that part? In fine, we cannot help feeling some degree of surprize that a physician of such talent and sense as Dr. Carson evidently possesses, should broach so wild, not to say dangerous, a procedure in this melancholy disease.

---

9. *Yellow Fever.** A solitary rustic "pent up in UTICA"—not Cato's UTICA, but a UTICA in the back woods of America, has published a paper in the "Plough Boy," a transatlantic periodical, which is re-published in our respected cotemporary of the north, and contains, in our humble opinion, more rational and just doctrines than some of those propounded by certain self-supposed *scavans* of Europe. Thirty-five years' observation and reflection induce Mr. Coventry to believe that yellow fever is of local origin—"depends on vegetable putrefaction, and in that short stage which generally attends the fever, is not contagious; but if protracted, and assuming a typhoid shape, (as he believes both bilious and inflammatory fevers may and often do,) *the patient may generate an atmosphere around him which, without due care, may induce an indisposition in the attendants.*" But, says this judicious observer, "remove the patient to a healthy and elevated situation—keep him clean, and well aired, and I should expect no more danger from this disease than from a tertian intermittent." Our readers are aware that these are the doctrines long advocated in this Journal.

---

* Mr. Coventry.—Ed. Journal, No. 71.

10. *Pulmonary Disease.** Baglivi's remark on the great difficulty of distinguishing the various diseases of the lungs must have been forced on the mind of every person who goes through even a very moderate share of practice. The following case is one of those that are not a little puzzling.

" Agustine Hemon, a female, 36 years of age, of feeble constitution, and very nervous temperament, entered La Charité, first time, in November 1814, with all the appearances of peritonitis, and went out convalescent in the succeeding February. She experienced, however, at her departure shooting pains in the thighs, and dull pains in the abdomen, with bad digestion, and occasionally vomitings. From this time till her re-entry on the 11th July, Hemon experienced sharp and frequent pains in the loins, region of the uterus, and head. She had troublesome cough, and an habitual constipation was changed to a diarrhœa, which had lasted six weeks. There was œdema of the ankles—flushings of the face, pain in the nipples, heat in making water, shortness of breath, especially in going up stairs. In the month of April, she had experienced shiverings, succeeded by well-marked pyrexia and perspiration in the evenings. On entering the hospital on the 11th of April, there was considerable emaciation, with pains in the head, tinnitus aurium, troubled sleep, inability to lie on her back or left side, frequent startings in the night, breathing short, and interrupted by sharp thoracic pains, so as to be threatened sometimes with suffocation. The cough was not very severe, and came on in paroxysms, with scanty and difficult expectoration of mucus, sometimes streaked with black blood. Muscular motion was attended with great anxiety and tendency to syncope. She felt shooting pain in the region of the heart extending to the spine, with sense of great oppression at the pit of the stomach, and tenderness on pressure in both hypochondria. Constipation now had succeeded diarrhœa, with sometimes a discharge of blood from the rectum. Skin was hot, and fever pretty constant, with morning perspirations. Emaciation now made rapid strides. The dyspnœa and anxiety increased daily. She was bled, but this measure produced no relief; and the same want of success attended the administration of antiphlogistic, antispasmodic, and revulsive remedies. The patient died at the end of three weeks from her entering the hospital.

" On dissection, the pleura, heart, pericardium, peritoneum, and all the abdominal viscera were found perfectly healthy. But the lungs were observed to be filled with small vesiculo-cartilaginous bodies, the size of millet seed, which, however, did not prevent the intermediate parenchymatous structure from being perfectly permeable by the air, and crepitous. The mucous membrane at the lower extremity of the œsophagus was red, and covered with albuminous filaments." P. 374.

---

* Dyspnée Nerveuse febrile, et Degeneration Vesiculo-Cartilagineuse du Tissu Pulmonaire. Par. M. Fouquier. *Annuaire Med. Chir.*

Such, says M. Fouquier, was the history of a disease which presented, during life, the phenomena of various phlegmasiæ, "but which were merely nervous." Death could not, he thinks, have been caused by the pulmonary degeneration of structure. "Was this painful and promptly fatal dyspnœa to be attributed to asthma?" We cannot but attribute the functional disorder in the chest and various other parts of the body to the structural change in the lungs, notwithstanding the contrary opinion of M. Fouquier. The respiratory function, and especially sanguification, could not be otherwise than materially molested by such a state of the pulmonary structure; and the influence of such molestation on the various other functions of the system may be readily appreciated.

---

11. *Oil of Croton-Tiglium.* This medicine, we have every reason to believe, will prove a valuable addition to our materia medica. Mr. Conwell, lately returned from India, has brought thirty or forty quart bottles of the genuine oil with him, and therefore there will be an ample supply in the market. We believe he means to bring it under a stamp, and then every one may be sure of having the unadulterated substance.

We have had some opportunities of administering this remedy, and we have seen many medical men who have used it on a large scale. It certainly does produce nausea in several instances; but we have no doubt that means will be found to correct this quality of the oil. Dr. Nimmo of Glasgow has published some chemical and therapeutical observations, in the April number of the Royal Institution Journal, on the composition and qualities of the seeds of the croton-tiglium, from which we shall extract a few particulars.

Having procured a very small quantity of the oil, (12 drops) he poured on it two drachms of alcohol, which produced a partial solution. Pouring this off, two drachms more of alcohol were added, by which an additional portion was dissolved. A third quantity seemed to have no effect. There remained an oily-looking substance, equal to somewhat more than a third of the original oil.

The alcohol solution exhibited the characteristic acrimony of the oil—the undissolved portion had none whatever. The solution he considers as preferable to the entire oil, obviating the following objections—namely, the difference of dose in consequence of the inequality in the thickness of the lips of phials—the greater or less degree of viscidity in the oil at different temperatures—and the difficulty of apportioning the dose to difference of age or constitutional susceptibility to the action of the ordinary purgatives. In administering the alcoholic solution in doses relative to the number of drops decomposed, the same effects were produced as have been attributed to the entire oil.

Dr. Nimmo has made a great many analytical experiments on the seeds and kernels of the croton, extracting from the latter an oil equal, if not superior to that prepared in India. But as the original oil is now plentiful in the market, we shall not stop to notice these chemical analyses. Dr. Nimmo recommends the alcoholic solution of the oil to be so prepared, that half a drachm of it may contain a drop. It may then be administered in the following manner:—℞. Alcohol. croton. ʒss. syrupi simplicis, mucilag. accacæ āā ʒij. aq. distillat. ℥ss. m. ft. haustus. "After swallowing a little milk, take the draught very quickly, and wash it down with repeated quantities of the same diluent." He has administered this remedy in more than one hundred instances, and to some patients many times. In not more than three or four cases was vomiting produced—and that not in a violent degree—in not many was nausea felt—in all cases purging was induced in a space of time between half an hour and three hours after taking the medicine—the purgative effects were generally moderate, and rarely accompanied by griping. He attributes these steady and quiet effects to "the instantaneous and equable diffusion of the active principle of the croton over the inner coat of the stomach and abdominal viscera, when the above formula was employed." Whereas, he observes, it must unavoidably happen that the oil, taken merely mixed with any fluid; made up into pills with any substance; or diffused with sugar or starch, may, at times, be applied in a concentrated form to a particular part of the stomach or intestines, and excite nausea and vomiting in the one case, and spasmodic action, with pain and hypercatharsus, in the other.

Among the cases which Dr. N. had occasion to treat, was a lady, who, for the cure of abdominal dropsy, had undergone a course of mercury, and used diuretic medicines of the most powerful kind, without effect. She was rapidly sinking under an accumulation of the dropsical fluid, with a total loss of appetite and strength. The alcoholic solution of the croton was administered, at first cautiously, and afterwards in augmented doses, so as to cause three or four evacuations daily from the bowels, with the most beneficial consequences. By augmenting the appetite and strength, and by the discharge of watery stools, the size of the abdomen was soon reduced. After two weeks' use the stomach became irritable, and the croton was suspended. The complaint shewed strong symptoms of returning—diuretics again failed; and again recourse was had to the croton combined with opiates and aromatics. By this the cure was effected.

Our author found the croton a powerful auxiliary to opium in the cure of delirium tremens. "In all cases in which there is a superfluity of the secretion of bile regurgitating into the stomach, it is of service, unless the medicine itself be rejected by vomiting." In the removal of jaundice from obstruction or spasms in the bowels, it proved most beneficial. "In a case of excessive corpulence, with the most alarming symptoms of determination to the head, amounting at times almost to apoplexy, a few doses of the solution produced the most signal benefit; and, without having had occasion to let blood, every symptom was removed which could have been expected from venesection."

We hope the attention of the faculty will be directed more strongly than ever to this powerful medicine. We think it will prove a valuable adjunct to other purgative substances, when their operation is wished to be quickened. We have advantageously combined it, of late, with aloes, quicksilver pill and squill, in two cases of dropsy; and in three or four other cases we have observed its power of increasing the action of the kidneys.

---

12. *Bath Waters.** An anonymous writer in the Journal of Science deplores, in very feeling terms, the neglect into which the once celebrated waters of Bath have now fallen. He attributes the public depreciation of these waters, in a great measure, to the writings and opinions of the late Dr. Parry. This anonymous writer makes Dr. Parry to state that all diseases, *without exception*, consist "either in absolute or relative excess of momentum, impetus, or determination of blood in some portion of the arterial system." Now our readers have only to turn to page 36 of this volume to be convinced that this anonymous critic (like many critics of the present day) never read the work he censures. True it is, that Dr. Parry, (as well as every other observant physician) traces the majority—the great majority of diseases to partial or general increase of circulation; but he does not make this a universal law in pathology. We have noticed in our review of his work several of those diseases which that eminent and enlightened pathologist attributed to a state the reverse of what is quoted above.

Mr. Abernethy is next assailed, and the "deranged digestive organs" ridiculed, like the vascular doctrines of Dr. Parry.† The anonymous writer is somewhat more happy in his hits at a celebrated medical surgeon in Bath, whose practice forms a kind of antithesis to the old Bath-water courses. Instead of pouring down fluids by the mouth, this gentleman exhibits solids *a tergo*—that is by the tail—a *fundamental* kind of practice which the writer ap-

---

* Mr. Brande's Journal, No. XXV.

† The Abernethian School might quote very ancient medical authority for the *gastric* doctrine of their master. Among others, *Serenus Samonicus*, who, in his medical precepts, has the following remarkable passage, which might serve as a very excellent motto for the school in question.

"Qui stomachum regem totius corporis esse
"Contendunt, vera niti ratione videntur.
"Hujus enim validus firmat tenor omnia membra:—
"At contra ejusdem franguntur cuncta dolore.
"Quin etiam (nisi cura juvat) vitiare cerebrum
"Fertur, et integros illinc avertere sensus."

*De Med. Precept.*

Can this piece of antient pathology be questioned or excelled in the present day?—*Ed.*

pears to consider " most disgusting and dangerous," though (strange to say!) " accompanied with an appearance of satisfaction, not unlike that of the courtiers of Louis XIV. who, by a strange perversion of sentiment and loyalty, were not only proud to be the favourites of the Grand Monarque, but of being likewise counted martyrs to the same disease with which that personage is known to have been afflicted." It is hardly necessary to say, that we are far from encouraging *peculiar practices* in medicine. Diseases are so proteiform, and constitutions so dissimilar, that various and often opposite remedies, must be employed in the same complaint. In fact, the great art of medical practice, consists in watching the phenomena exhibited by the constitution and the disorder; and adapting the remedies to the existing state of the case.* By the anonymous writer's account, such is not the rule at Bath; for on looking over a file of prescriptions in a chemist's shop there, " twenty-eight out of thirty consisted of the blue pill."

---

13. *Ascites cured by Pressure.*† It is but very seldom that we see abdominal dropsy cured, after the operation of paracentesis. The following case is therefore interesting.

" Mary Mattan, 21 years of age, had enjoyed good health, and menstruated regularly till within six months of the date of this report. She entered the Hotel Dieu on the 23d January, 1815. During the preceding half year, and without any known cause, her abdomen daily enlarged in size, without pain or any kind of indisposition. Latterly, her breathing became embarrassed, her appetite depraved, her sleep interrupted, and her spirits depressed. Examined with care, she presented the following phenomena:—considerable emaciation—abdomen very large, not painful, but offering on percussion, unequivocal fluctuation. Diuretics of squill, nitre, and digitalis—purgatives—diluent drinks. The urine was increased, and the size of the abdomen diminished. But this success was only ephemeral, and the dropsy gaining ground, recourse was had to paracentesis. M. Dupuytren drew off a considerable quantity of limpid serum. The abdomen was then carefully examined, but no viscus could be found organically affected. Resin of jalap and nitre were then administered in a diuretic drink. The secretion of urine increased at first; but the abdomen began to enlarge again, and fluctuation was soon evident. This was on the 20th April. Abdominal compres-

---

* A curious instance of idiosyncrasy was recently witnessed in the person of a medical gentleman, (Mr. Crawford, late of Southampton,) who, while labouring under phthisis, was advised to try the prussic acid. He took a mixture containing only three drops, which raised the pulse from 96 or 100, its usual rythm, to 150, and it never fell below this afterwards, till his death. We only saw him a few days before his decease, and his first words were—"I have been poisoned by prussic acid."

† M. Husson. *Annuaire Medico-Chirurgical.*

sion was now determined on. A bandage was well applied, and drawn tighter as the size of the abdomen diminished. Under the influence of this new measure the urinary secretion increased—the volume of the abdomen diminished, and all sense of fluctuation soon disappeared. On the 6th of May, the patient left the hospital completely cured, and has since retained good health."

It is needless to observe that, bandages are always applied after paracentesis abdominis; but rather with the view of supporting the abdominal viscera after the removal of the water, than from expectation of their preventing effusion, or promoting the absorption of a fluid effused. They are not, in general, applied with that degree of tightness, or kept uniform enough to answer the latter purposes. The measure is, therefore, deserving of attention.

---

14. *Ovarian Dropsy cured by Operation.** This was a very bold operation, and verified the proverb—fortuna favet fortibus. We shall give the history of the complaint, and the operation in our author's own words.

" Mrs. Strobridge, aged 33. Seven years before, she perceived a small tumour in her right side, situated in the right iliac region; when about the size of a goose egg, she could move it with her hand to the opposite side of the linea alba, and to some distance above the umbilicus. The patient had borne five children, two previous, and three subsequent to her discovering the tumour. The youngest child was 10 months old, and was nursed at the breast when she submitted to the operation. Soon after her first pregnancy, from the commencement of the tumour, and when, as she thinks, it was about 4 or 5 inches in diameter, it suddenly disappeared, probably burst into the abdomen. In 4 or 5 weeks it was as large as before. Before and after the bursting of the tumour she had turns of faintness, which lasted from two hours to half a day. During parturition of her second child, after the commencement of the tumour, it having acquired a considerable size, it burst again, and nothing was perceived of it till eight months had elapsed. In four days from its reappearance it was as large as it had ever been. It was again burst by a fall; great soreness of the abdomen, and confinement of the patient for several weeks was the consequence. The tumour filled again in a fortnight, and from this time continued to increase; it did not burst in the delivery of her last child, which was ten months previous to the operation. The patient's health was not much affected by the tumour. She was costive; and the size of the tumour incommoded her in the ordinary duties of her family, especially in stooping. On examination I found a large tumour in the right side of the abdomen; it was considerably moveable, and I could produce a distinct fluctuation through it.

---

* Amer. Med. Recorder, No. 17.

"Having decided on the operation, and determined the mode of operating, on the 5th of July, in the presence, and with the assistance, of Doctors Lewis, Mussy, Dana, and Hatch, I commenced the operation as follows:—

"The patient being placed on a bed, with her head and shoulders somewhat raised, an assistant rolled up the tumour to the middle of the abdomen, and held it there. I then commenced an incision about an inch below the umbilicus, directly in the linea alba, and extended it downwards three inches. I carried it down to the peritoneum, and then stopped till the blood ceased to flow, which it soon did. I then divided the peritoneum the whole extent of the external incision. The tumour, now exposed to view, was punctured; a canula introduced, and seven pints of a dark coloured ropy fluid was discharged into a vessel; about one pint was spilt, so that the whole fluid was about eight pounds. Previous to tapping the tumour, by inserting my finger by the side of it, I ascertained that it adhered to some extent to the parietes of the abdomen, on the right side, between the spine of the ilium and false ribs. After evacuating the fluid I drew out the sack, which brought out with it, and adhering to it, a considerable portion of the omentum. This was separated from the sack with the knife; and two arteries, which we feared might bleed, were tied with leather ligatures, and the omentum was returned. By continuing to pull out the sack, the ovarian ligament was brought out, this was cut off, two small arteries, secured with leather ligatures, and the ligament was then returned. I then endeavoured to separate the sack from its adhesions to the parietes of the abdomen, which occupied a space about two inches square; this was effected by a slight stroke of the knife at the anterior part of the adhesion, and by use of the fingers. The sack then came out whole, excepting where the juncture was made, and I should think it might weigh between 2 and 4 ounces. The incision was then closed with adhesive plaster, and a bandage applied over the abdomen. No unfavourable symptoms occurred after the operation; in three weeks the patient was able to sit up and walk, and has since perfectly recovered.

"I was induced to undertake this operation from the following considerations:—The patient, though her health was not greatly impaired, was sensibly affected by the disease. She was quite certain that the increase of the tumour, in a given time, was augmented; probably, at no very distant period, it would destroy her. I had, also, an opportunity to dissect the body of a patient, who had died of ovarian dropsy, after being tapped seven times. In this case the sack was found to be in the right ovarium, which filled the whole abdomen; but it adhered to no part except the proper ligament, which was no larger than the finger of a man. I have seen two other ovarian sacks which were taken from patients after death. They had been tapped several times; the sacks were equally unattached, except to their own proper ligaments. Hence, I inferred, that in a case of ovarian dropsy, while the tumour remained moveable, it might be removed with a prospect of success. The mode of operating, practised in the above case, is the same as I have described to my pupils in several of my last courses of lectures on surgery. The event has justified my previous opinions.*"

---

15. *Poisoning by Arsenic.*† A young married woman swallowed a quantity of arsenic near midnight, and next morning, at 8 o'clock, was found by Mr. Hume suffering the most excruciating torments, constant efforts to vomit, and "all the symptoms peculiar to the arsenical virus." It appeared that she had vomited once or twice rather copiously. Mr. Hume instantly prescribed the following mixture, viz. an ounce of carbonate of magnesia, a drachm and a half of vinum opii, three drachms of sp. lavand. comp. half an ounce of sugar, and sixteen ounces of distilled water. Two large spoonfuls were directed to be taken every ten minutes, while the symptoms continued violent. The first bottle produced a remission of the symptoms, and the second bottle seemed to have effected a cure. It is to be regretted that Mr. Hume's avocations should have prevented him from ever visiting the patient after the first time, till she came to him on the 5th day after the accident.

What share the magnesia may have had in counteracting any arsenic that might have been remaining in the stomach, or the *effects* of the arsenic, if already disgorged, we leave to the determination of our readers. For our own parts, we are rather sceptical on this occasion, since the case is hurried over, and brought at last to a somewhat impotent conclusion. Mr. H. however, has promised farther particulars, and to these we shall revert, if they appear in time.

P. S. In the succeeding number, Mr. Hume resumes his narrative; but *now* the cure is attributed to magnesia and *opium*. In this second paper our author flies from subject to subject with such astonishing rapidity, that we frequently lose sight of him entirely; in short we are quite incapable of deciphering what Mr. Hume would be at. We cannot but join with Mr. Hume in the following sentiment:—"I regret that this woman's case had not fallen into other hands, and that more attention on my part could not be bestowed to render it more complete. I visited her not more than three times, and these were not daily; so that the progressive state of the pulse and many other particulars are evidently wanting." We have the highest respect for Mr. Hume as a scientific chemist, but his best friends will acknowledge that he makes no great figure out of his own department.

---

* The author, at the time of reporting the above case, was ignorant that Dr. Dzondi had proposed the attempt to cure ovarian dropsy by the introduction of a tent following puncture, that the dropsical sack might slough, and be withdrawn by forceps.—Vid. Medical Recorder, Vol. III. p. 63.

† Mr. David Hume.—Lond. Med. Journ. 273

16. *Tympanites.** Professor Liquiere informs us that it was the successful treatment of this case that first brought him into notice at Autun, and laid the foundation of his renown, at a time when a host of circumstances combined to render his establishment in practice almost hopeless. Our readers will naturally be curious to know what this tide was that led him on to fortune. It was as follows.

A man, 30 years of age, and previously healthy, was seized, in the year 1814, with violent colic, obstinate constipation, great eructation of wind, considerable distention of the abdomen, the violence of the pain leaving him no intervals of repose. In a day or two, however, the constipation gave way spontaneously—a diarrhœa came on—and the complaint disappeared. At the end of a few days the colic returned, and the pain was more violent than ever. Various means were now tried for the removal of the complaint, as purgatives, liniments, potions, lavements, baths, blisters, &c. but without effect. Our author was then called in, the patient having been ill 22 days, during which no passage by stool had been procured. Dr. L. found the patient meagre and emaciated, the eyes hollow and expressionless, tongue dry and red, no thirst, great difficulty in swallowing, breathing free, great eructation, constant crying out with pain, pulse feeble but not frequent, the whole body in a state of marasmus except the abdomen, which was swelled and tense as a drum, very little sensible to pressure, and sonorous on percussion; the intestines so inflated that their course could be distinctly traced through the integuments; borborygmi so loud that the neighbours were firmly persuaded some living animal was in the patient's abdomen; constipation of 22 days standing; nourishment one cup of broth daily. The patient had made several attempts to destroy himself.

Dr. L. here falls into a long train of reasoning on this case, or as he terms it, "*a mental analysis*" of the pathological elements that entered into its composition. The primitive elements he at last reduced to two—pain and spasm. He makes it out (to his own satisfaction at least) that of these two the pain was the original and the spasm the consequence of pain. This is curious reasoning, for what is pain but the sense of some other morbid state? Is it not far more likely that the pain was the consequence of spasm? Be that as it may, he hit on the right therapeutics. He gave the patient, on the spot, a grain and a half of opium. This was at noon; and at one o'clock the dose was repeated, which removed the pain like a charm, and threw the poor fellow into a sound sleep of three hours. On awaking the pain returned, but with less violence, the constipation and abdominal tension remaining the same. The opium was repeated, and again the patient fell into a profound sleep. In the morning he complained not of pain; and now a purgative injection of salts, manna, &c. was administered. Presently the patient felt an inclination to stool, and now "la scène excrementitielle com-

* Professor Liquiere. Journ. Compl. Oct. 1821.

menca." The patient sent forth such prodigious quantities of flatus and feculencies, that the neighbours assembled to view the miracle. It is almost needless to state that from this moment the man was freed from all complaint.

Every good practitioner in this country would, in such a case, have pursued nearly the same course—excepting, perhaps, that he would have combined opium and purgatives together, a most excellent and judicious practice in a great variety of complaints, whatever may be said to the contrary by speculative physicians.

---

17. *Bronchocele** The patient was affected with a considerable enlargement of the thyroid gland for 20 years, together with a tumour on the left side of the neck, distinct, in some measure, from the bronchocele. The common carotid artery was found pulsating strongly immediately under the platysma myoides, and could be grasped by the finger and thumb. The vessel appears to have been displaced by the lateral tumour. The superior left thyroideal artery was also found pulsating strongly in the upper part of the tumour. The patient suffered considerably in swallowing and in speaking sometimes. Our author determined on taking up one or more of the thyroid arteries. The operation was performed on the 10th of May, 1821. Having reached the thyroid artery by a cautious dissection, an animal ligature of suitable size was applied. The incision healed kindly over the ligature. The patient being of a delicate nervous constitution, suffered considerably for some days, from tremblings, chills, giddiness, and anorexia. These symptoms gradually subsided; and, from the time of the operation the patient "had more freedom in swallowing," was less teased with a drawing sensation under the eye, and she could lie lower down in bed. In a few weeks after the operation the tumour was evidently smaller, less painful, and hung looser and lower down. "In short, the operation has been greatly useful, and will probably prevent any further growth."

---

18. *Arteritis.*† The subject of this disease was a young man, 32 years of age, of delicate constitution, and very limited intellectual powers. About three years ago he became melancholic, and fond of solitude, which ended in mental derangement. After having been nine months under the care of Dr. Esquirol in his *Maison de Santé*, he was conducted to the Charenton, on the 19th February, 1819, where he remained five months without any amelioration of the mental hallucination. He was thin, but apparently in health

---

* Dr. Jameson, American Med. Recorder, No. 17.

† Observation d'Arterite, Par M. Bayle, Interne en Medecine à la Maison Royale de Charenton. Bibliotheque Medicale, Sept. 1821.

of body. During the last two or three months, the patient experienced some difficulty of breathing, which gradually increasing, was attended with œdema of the feet. Five days after this he was examined by the reporter, and the following phenomena were noted. Face flushed—lips of a violet colour—sense of great fatigue and weight on attempting to move—anxiety—general malaise—hands violet colour—abdomen tense, and apparently containing an extravasated fluid—feet cold and œdematous—respiration quick, laborious, and difficult—pulse small and frequent—pulsations of the heart strong and irregular—no appetite—constipation of the bowels. Diuretics and diluents prescribed. In a day or two he became clear in his intellects, and related in the most distinct and faithful manner, the symptoms of his complaint. All the symptoms above stated continued to increase, and Dr. Royer-Collard being consulted, pronounced the disease to be an affection of the heart, and probably of the large vessels. Died three days afterwards.

*Dissection.* The arachnoid membrane opake in several points—thickened and very resisting, particularly on the sides of the cerebral hemispheres. Pia mater infiltrated with a serous fluid, which was found in considerable quantity also at the base of the brain, and in the lateral ventricles.

*Thorax.* In each cavity of the pleura about half a pint of serous effusion. Lungs sound—heart twice and an half its natural size—the right auricle prodigiously dilated, and containing clots of black blood—and its internal surface covered with a molasses-like substance apparently different from the fibrine of the blood. The right ventricle was also much dilated, and its carneæ columnæ remarkably strong. The parietes of this chamber were double their ordinary thickness. The inner coat of the pulmonary artery was of a scarlet colour throughout, which appearance could not be scraped off by the scalpel. The interior of the left auricle presented the same phenomenon as the right; except that the lining exudation was of greater consistence and yellower than in the last-mentioned chamber. The lest auriculo-ventricular opening was contracted, so that the point of the little finger could scarcely be passed through it. The left ventricle was dilated in respect to cavity, and thickened in regard to parietes. The aorta was smaller than the pulmonary artery, and not above two thirds of its natural diameter. Its inner coat presented the same scarlet appearance as the pulmonary artery; but the venæ cavæ were natural.

There was some effusion in the cavity of the abdomen—the mucous membrane of the stomach was reddened—the liver enlarged—the intestines sound.

The above case we consider to be interesting, both in respect to the pathologia of insanity and cardiac affection. From what we have seen ourselves, and learnt from those who have the charge of lunatic asylums, the brain very generally presents the appearances above described in the malady in question. We consider the patient, in this case, to have died from disease of the heart as much as from inflammation of the arteries.

Mr. Smerdon* has related an interesting case of pulmonic inflammation, terminating in (we should prefer saying, accompanied by) arteritis. The pneumonia was most obstinate, and accompanied, from the beginning, with pain, in the *left hypochondrium.* Decisive depletion relieved the symptoms, but a relapse took place, with sense of pressure below the thyroid cartilage, difficulty of swallowing, loss of voice, sense of fulness, pain, and *pulsation in the left hypochondrium.*† In two days from this time the patient died.

*Dissection.* Twenty-four ounces of bloody serum in the left side of the chest—bronchial ramifications filled with a thin colourless fluid—substance of the lungs soft, and easily crashed by the gentlest pressure. The heart was flaccid, but the interior tunic of the aorta, as far as it could be cut out with a scalpel, was affected with the most intense inflammation, being much thickened, hardened, and easily stripped off with the nail. The pulmonary artery was natural.

We do not see just reason for Mr. Smerdon supposing that the pulmonic inflammation terminated in arterial. He says "the pulsation in the epigastric region was symptomatic of the arterial inflammation." But the patient complained of pain there long before the relapse, and he does not appear to have been examined with the hand till the 21st, a day or two before his death. Then, of course, the *pulsation* was ascertained. It even appears to us that the immediate cause of death was the effusion both into the bronchial cells, and the thoracic cavity—at least this effusion was quite sufficient, independent of the arteritis, which we cannot look upon as more than an accompaniment—not a conversion of the pulmonic inflammation.

We regret that the examination of the arterial system was so limited. The arteritis appears to have been discovered after the heart had been removed, and the body sewed up.

Mr. Smerdon makes some interesting observations on pulsation in epigastrio resulting from irritating matters in the stomach, or accumulations in the bowels, which we recommend to the attention of our brethren.

---

* Med. and Phys. Journal, Dec. 1821.

†"In our article on arteritis, in a former number of this Journal, we alluded to Aræteus as seeming to have known something of the disease under consideration. The following passage is quite unequivocal, and which we did not then observe.

"Venæ concavæ, crassæque arteriæ, quæ secundum dorsum extenduntur, inflammationem ardoris speciem majores nostri appellaverunt; nam ardoribus similes affectus in ambabus oriuntur; ignis acutus, et acer, fastidium, sitis, anxietas, *pulsus palpitans in præcordiis* et in aversa parte, quam græci METAPHRENON vocant et quotcunque a me in volumine de signis relata sunt," &c.—*Lib.* II. *Cap.* 7.

Aræteus advises very active treatment in this serious disease. Venas itaque in cubito cedito, multumque sanguinis, sed non semel totum mitteto, immo et bis, et ter, et alio die, quo interim vires instaurentur repetito." *Ib.—Ed.*

19. *Inflammation of various Organs.** The darkness of French therapeutics is every day throwing a blaze of light on pathology—in other words, the inefficiency of the remedial measures employed by our neighbours, is daily corroborating, and indeed improving, the rational and energetic practice of this country. We hope a day will come, when our Gallic brethren shall open their eyes to the errors which they are now committing. Their conduct, indeed, in this respect, is one of the many enigmas exhibited by that ingenious but excentric nation.

*Case.* Jean Croquois, a fusileer, 19 years of age, of bilio-sanguine temperament, and enjoying good health till the month of January, 1820, committed about that time several excesses, particularly in spirituous liquors. On the 5th of February he was taken ill, and entered the Val de Grace Hospital, complaining of intense heat internally, ardent thirst, pulse quick and hard, skin hot and dry, disgust for food, slight pain in the abdomen, violent head-ache, pains in the lower extremities, disposition to shiverings. *Thirty leeches to the epigastrium—gum water edulcorated.*

6th. Next day the symptoms had preserved their intensity, and there was, in addition, a severe and frequent cough. *Edulcorated gum-water continued—a poultice to the chest—a venesection of twelve ounces.*

7th. Slight diminution of the cough—but the pulse is still frequent and full—tongue red and dry—thirst intense. These symptoms continued without diminution till the sixth day of the disease, (10th) when abdominal pains and diarrhœa induced to the application of leeches to the anus, which checked the colic and looseness, but produced no mitigation of the general inflammatory symptoms, before described.

15th. Delirium during the night, with subsultus. In the morning a reduction of cutaneous temperature—swelling and lividity of the feet—piercing cries, and fierce expression of countenance. *Gum water, warm cataplasms to the feet.*

18th. General prostration of strength—cough teazing; but no expectoration—stools involuntary. Death at two o'clock in the morning.

*Dissection. Head.*—Vessels of pia mater and tunica arachnoidea injected—yellow serous infiltration between the lamina of the arachnoid, and between this membrane and the pia mater. The pia mater and arachnoid covering the anterior superior part of the cerebellum very much thickened, and in some places suppurated. The substance of the brain was soft; the ventricles distended with about an ounce of water; the plexus choroides pale and infiltrated; a serous effusion in the cavity of the spinal chord.

*Thorax.* Lungs red and containing much blood, but still a little

---

* Scouttetten, Surgeon to the Military Hospital of Val de Grace. Journal Universel des Sciences Medicales, No. 68.

crepitous. The mucous membrane of the larynx and trachea intensely inflamed. The heart appeared more voluminous than natural, and the parietes of the left ventricle were nearly an inch in thickness. In this chamber, near the aortic orifice, there appeared an ulcer about an inch in diameter, the edges of which were swelled, red and ragged.

*Abdomen.* The stomach and intestines viewed externally appeared sound; but, on more accurate investigation, there were several portions of the digestive tube contracted, red, and inflamed. The mucous membrane of the stomach was pale, excepting a few points near the pylorus. The interior surface of the intestines presented several points of inflammation. Liver of a deep red colour and gorged with blood. Other viscera sound.

The author of this narrative, with great self-complacency and sagacity, amuses himself—and the society where it was read, with remarks on the correspondence between the phenomena during life and the appearances on dissection, without ever dreaming that he was himself most highly culpable in looking on at this unequivocal index of internal inflammatory devastation, prescribing his gum-water and leeches, when he ought to have been bleeding to syncope, or till the cessation of those symptoms which he so coolly describes.

There is not a surgeon-apothecary's assistant in the British dominions who would not be turned out of his situation for such gross misconduct. It is on this account that we can only have *graphic descriptions* of such exquisite pathological specimens in this country. The originals cannot be seen here—for who would have the courage to acknowledge himself the medical attendant, where such terrible proofs of his incapacity appeared on dissection? But, as we have often before observed (and cannot too often repeat) these specimens are most invaluable beacons to keep us in the safe channel of practice where we now are, and warn us of the dangers which lurk under half measures in acute diseases.

---

20. *Identity of Variola and Varicella.* Mr. Bampfield we know to be a gentleman of integrity and discrimination; we should, therefore, be inclined to place confidence in his statements. The case in question bears immediately on the important question now agitating the professional world.

Mrs. D. had been vaccinated when young. On the 14th Dec. 1821, she became affected with pyrexia, head-ache, and nausea. On the 16th Mr. B. saw her, when an eruption of small red protuberances, differing slightly in size, and nearly circular, with a transparent vesicle in the centre, had spread over the face, neck, breast, arms, and hands. He pronounced the disease varicella. On

---

* Mr R W Bampfield. Med. and Phys. Journ. No. 278.

the 17th the eruption had spread all over the patient, and was painful in the palms of the hands. On the 18th the febrile symptoms had considerably abated, and the lymph in the vesicles had become slightly straw-coloured. On the 19th the vesicles on the face began to pucker and fade, this stage being less observable in other parts. On the 20th there was no fever—a small brown scab had begun to appear on some of the vesicles; which, on the 21st, had become general, and fell off from the 8th to the 11th day. Mrs. D. punctured the vesicles on the palms of the hands, which had the effect of entirely evacuating the contained lymph, and of leaving them on a level with the surrounding integuments, without being again filled.

Her infant on the breast, which was two months old, *not vaccinated*, shewed, on the 25th December, numerous little red spots on the face, neck, and breast. His eyes were red, his throat sore, and the febrile symptoms severe. The eruption gradually spread to the extremities, and some eruptions appeared to come out successively on parts of the body, for two or three days. On the 26th the lips, the tongue, and the membranous lining of the mouth and fauces were thickly studded with the eruption, occasioning great difficulty in sucking and swallowing. The pocks were distinct—surrounded by a circular margin of inflammation—and gradually filled and enlarged. The fever became moderate; but the irritation and restlessness were alarming. "On the 5th day it was evident the disease was not varicella." "The most superficial observer would now have pronounced the eruption to be small-pox." The central depression was evident; "and it was discovered that the structure of the pock was cellular, and filled again on being punctured." The child died on the 8th or 9th day of the eruption.

On the day of the child's death, Master Dickenson, a younger brother of the mother, and who had been living with her during the period of her sickness (formerly vaccinated) became ill. On the next and succeeding days an eruption came out, exhibiting transparent vesicles in the centre. "This eruption was similar to Mrs. Drury's, and followed the same course." On the 7th day the boy was so well that Mr. Bampfield withdrew his attendance.

We are at a loss to conceive on what grounds Mr. Bampfield upholds the identity of variola and varicella, as far as these cases are concerned. We do not see a single particle of evidence in the whole affair that tends to this identity. Mrs. Drury *had* been vaccinated—and she exhibits what have been described as *modified* small-pox. Her infant had *not* been vaccinated—he exhibits unequivocal variola, and dies. Master Dickenson, on the other hand, had undergone vaccination, and he goes through the modified form, the same as Mrs. Drury. What has varicella to do with the business? As for the *cellular structure* in the infant's case, Mr. Bampfield will hardly set up a distinction as a proof of identity. There appears, in our humble apprehension, nothing in these cases but proofs of the modifying power of vaccination. We do not enter into the general question of the idendity of variola and varicella at all—we only comment on the evidence before us in Mr. Bampfield's paper.

21. *Inflammatory Dropsy.** The writings of Blackall, Crampton, Parry, Abercrombie, and many modern authors, have thrown much light on the nature, and improved the treatment of dropsy. We were lately much pleased with some observations of Dr. Graham, in the 71st number of the Edinburgh Journal, as they conincide with our own opinions, and will, we hope, prove useful to the rising generation of the profession. The professor very properly observes, that large and small doses of medicine or detractions of blood are merely relative terms, and should never be understood as denoting absolute quantities—for what would prove a large dose or detraction in one person, might prove trifling in another. The general rule of conduct, therefore, ought to be derived from the *sensible effect of our practice.* Every dose of medicine, however large, is too small—every quantity of blood drawn, however great, is too little, if it stop short of the usual sensible effect on the constitution, though not perhaps on the disease.

"If," says Dr. G. "we produce upon the constitution the effect we have reason to look for, and the disease remains as before, then we have indeed fairly tried the prescription, and have sufficient reason to conclude that our treatment is inapplicable. If we direct purgatives, and succeed in opening the bowels freely, without relieving the disease, then we have reason to think that cathartics are not suited to the case. If we employ blood-letting in a patient with a full hard pulse, however large the quantity may be that we take away, we do not try the remedy if we stop before we have produced its usual sensible effects,—before we have brought down the fulness and strength of the pulse; but if we have done this, and the disease continues unabated, then we are justified in changing the treatment." *Edinb. Journal, No.* 71, *p.* 226.

This we are convinced is the proper general rule of conduct—but, of course, there are many exceptions to all general rules in medicine, and the good physician will not act, like an automaton, upon an invariable principle, but adapt his practice to the *specialities* of the case.

In respect to blood-letting, the ultimate effect of this measure is to diminish the strength of the circulation, though in the first instance it very often raises it. When we determine on bleeding, where there is a hard bounding pulse, we may allow the stream to flow till the circulation is moderated. The case of inflammatory dropsy minutely detailed by the professor, forms a practical commentary of the best kind on the foregoing reflections.

*Case.* Robert Norris, a robust young Irish labourer, presented himself at the Infirmary, swelled from head to foot with anasarca, and having obscure fluctuation in the abdomen, little appetite, and thirst; bowels open from medicine; urine scanty; pulse 66, small, and soft. Three days before leaving work, had been attacked with

---

* Professor Graham, Edinb. Journal, No. 71.

severe rigors, sense of weight and heat at the epigastrium; after which the œdema commenced. He had only taken some purgative medicines. Dr. G. learned that he had been much overheated in digging, and as often chilled on going home from his work. The symptoms of fever being inconsiderable, Dr. G. tried the warm bath, purgatives, and diuretics, (calomel, squill, and digitalis,) and on the 3d day blood-letting to twenty ounces, without producing any beneficial effect. On the 4th day he was again bled. On the 6th day another bleeding to 32 ounces, when the pulse rose in frequency and became more soft. The crassamentum had a gelatinous coat, and was contracted on the surface. Seventh day, pulse 80, and soft; but still remarkably full. For several days the lancet was not had recourse to, and the œdema went on increasing, attended with some dyspnœa. The young man now presented a very aggravated case of anasarca, with ascites, and perhaps hydrothorax. "He was distended like a sack"—his weight and bulk prevented motion —eyes sunk and small—appetite quite gone—nights restless. It was determined to carry the bleeding farther. *Seventy-two ounces* were immediately taken—the first 18 ounces flowing moderately free, the remainder in a very full stream—the pulse continually rising in firmness, and not becoming soft till the very last. There was no tendency to syncope. The patient observed that there was no alteration in his feelings till 60 ounces were abstracted—after this he felt much more happy. There was a slight buffy coat on all the blood. In half an hour after the bleeding he could lie in any position. He slept uninterruptedly in the night, and every night after. Next day his respiration was free, and he declared he felt quite well. Another bleeding, however, of 32 ounces, became necessary a few days afterwards, in consequence of a sense of oppression. This blood was buffed and cupped. From this period the disease rapidly subsided. The bowels were kept very loose by calomel, gamboge, and aloes. The urine rose to eleven pints per diem—the pulse generally about 90, full and soft—no debility attributable to the bleeding, nor other unpleasant symptom, except a degree of chilliness. Some time after he was dismissed cured. It was curious, says our author, to observe how fully the kidneys did their duty after the depletion, without the exhibition of diuretics. When the patient first came into the hospital, the urine was a mere nothing—after the second bleeding it rose to eight ounces—and in the 24 hours preceding the third bleeding he passed two pints of water. On the day after the third bleeding it was two pints and a half—and previous to the great bleeding, it was three pints and a half. After that bleeding it soon got to eleven pints per diem.

We think this case is calculated to do much good. We have no doubt that debility is too often kept up or induced by what is termed *cautious* bleeding—that is, bleeding to a certain *quantity*, and not to the production of a certain and necessary *effect.* We hope Dr. Graham will pursue this and other subjects with that zeal and intelligence which we think are plainly discernable in his character.

22. *Burns and Scalds.** It appears from Mr. Stokes that the late Mr. Shute, of Bristol, was accustomed to recommend the application of spirit of turpentine, or some warmed spirits, at first, "on the principle of a cooling lotion; knowing that, as the pain and heat of the part subsided, what at first was a cooling lotion, would become, if continued, a stimulating dressing." After the first day, therefore, or earlier, he inculcated a change to a milder application—the linimentum terebinthinæ, which might be lowered, if necessary, with olive oil, or simple ointment. He also strictly limited the spirit of turpentine to the injured surface—and had no objection to lotions to the surrounding parts. Where the vital powers were much depressed, he gave cordials—in others, opium to allay irritability. He was very particular in keeping limbs in a state of extension and rest. During the suppurating stage, the following cerate (in use at the Middlesex Hospital) Mr. S. considers as unrivalled. "It seems to soothe while it imbibes the discharge."—℞. Empl. plumb. ℥j. ol. oliv. ℥jss. liquefac. simul, et adjice, assidue movens, cretæ pptæ. acidi acetici āā ℥ss. ft. ceratum.

---

23. *Influence of Chagrin on the Hepatic System.*† The agency of moral emotions on our physical structure has been observed in all ages and countries. We are no advocates for the *identity* of mind and matter; but every thing shews us the strong though inexplicable bond of connexion between body and soul—whence spring all the sympathies of mutual pleasure and pain—all the reciprocities of rest and action. We see every passion of the mind act primarily, and with greater or less influence according to their force, on the functions of the body. We see the quickened circulation *follow* the anger—the start *follow* the surprize—the swoon *succeed* the sorrow. In these instances, and a thousand others, it is manifest that the priority of action belongs to the mind. But the converse is not the less apparent in numerous instances. We see mental emotions rendered more vivid by the application of vinous stimulus to the nervous coat of the stomach—depression of spirits follow dyspepsia—irritability of temper result from quickened circulation in the brain—prostration of mental energy from antimony in the stomach, &c. &c. These are all familiar to the least attentive observer; but the peculiar and striking effects of mental emotions on the liver and its functions, have not been so generally described, or indeed remarked, as their frequency would authorize. Of the instance related by M. Husson, in the work before us, we shall present a brief outline in this place, as it is calculated to excite our sympathy, and also our indignation on more accounts than one.

"Frances Guidet, a female servant, 32 years of age, of strong

---

* Mr. Stokes. Med. Repos. p. 101

† M. Husson. Annuaire Medico-Chirurgical.

constitution, but much sensibility, had enjoyed good health formerly; but having paid the most assiduous and zealous attention to a sick mother and son, in the family where she resided, this faithful domestic, exhausted by watching and fatigue, experienced in her turn a slight indisposition, when those she nursed had recovered. Instead of giving the poor young woman an asylum under the roof of those who were so much indebted to her, she was turned out of the house, and forced to seek shelter in an hospital! This act of ingratitude made such a deep impression on the susceptible mind of the poor creature, that she fell into a state of profound chagrin. When interrogated at the Hotel Dieu, she could only answer by sighs and tears. The most attentive examination could only detect debility of body and dejection of mind. She said she should die of chagrin—a prognostic but too fully confirmed! The day after she entered the hospital her skin and eyes presented a slight yellow tint—the countenance being indicative of much internal suffering. The yellow tint became deeper—the region of the liver swelled—the prostration of strength became more considerable. The right hypochondrium now became painful, the pulse small, the respiration quick, the stomach affected with bilious vomiting, the bowels with diarrhœa of ill-conditioned matters. No remedies appeared to do good. The liver projected below the ribs—and felt hard—the patient could only lie on her right side, and experienced great pain and misery. Leeches were twice applied to the anus without effect —to which were added diluents and antispasmodics. A bowel complaint reduced her to a skeleton, and finally terminated her existence on the 25th day after her entrance in the hospital. The inspection of the body was objected to by the friends."

This case offers a good illustration of the influence of mental despondency and chagrin on the hepatic system in particular. We have seen a very great number of similar cases, and they were generally much more unmanageable and fatal than hepatic affections from other or physical causes. It was therefore well observed long ago by the celebrated Ballonius:—" Nos vos (inquam) in ipsis operibus artis experientia dedicimus eos omnes quos meror ægritudoque animi in morbum conjecerit, aut letaliter aut gravissime periculosissimeque ægrotare. Tanta est vis ægritudinis ipsius pathematumque animi."—*Consil. medecin. lib.* iii. It is needless to remark that, in the present instance, no means were taken to check the disease that were at all calculated to effect that object.

---

24. *Poisoning by Arsenic.** A diabolical wretch attempted to poison four people in one house, and succeeded in one case out of the four. The fatal case did not terminate till the sixth day, and consequently no poison was to be substantially detected in the dead

---

* Mr. Murray, Ed. Journal, No. 71.

body. Neither was any particle of the arsenic found among the food administered—therefore there were no medical proofs but the symptoms during life, and the appearances after death. What the circumstantial evidences of the witnesses were we are not informed; but it appears that the criminal was condemned, and, before execution, confessed to the administration of arsenic. It is therefore interesting to know what were the grounds of the medical evidence.

The arsenic was taken on the morning of the 19th of August, and the man did not present himself to the narrator till the evening of the 24th of the same month. The man stated that on the 19th his illness commenced with sickness, succeeded by thirst, head-ache, and ultimately vomiting, which symptom often recurred during the next four or five days. In the early part of this space, the man was heard to complain of pain in his stomach, eyes, throat, breast, and arms. He voided his urine frequently. Yet his illness had scarcely at any time confined him to bed. On the 22d he took a dose of Epsom salts, which operated. On the 24th he rode six miles to see Mr. Murray, for the first time, complaining of the following symptoms:—pain and heat in the region of the stomach and lower part of the chest; occasional uneasiness in the abdomen, and sometimes ineffectual efforts to go to stool; thirst; difficulty of breathing; heat and uneasiness in the throat, with hoarseness: soreness in the eyes, which appeared inflamed; shifting pains in the extremities, particularly the arms, which were unusually weak; great restlessness; anxious expression of countenance, pulse from 100 to 110 —not strong. A blister was applied over the stomach and lower part of the chest, and an opiate ordered at bed time. On the following day (25th) Mr. Murray visited him at his own house, and found him nearly as above. His countenance exhibited a disturbed and anxious expression; the redness of his eyes and hoarseness were increased; and Mr. M. this day observed on the palate and uvula, small roundish white acuminated prominences, seemingly the membrane covering the palate bones and velum pendulum, detached at the parts by a whitish liquid. This day an ounce of castor oil was administered. He died in bed that night, without being observed. We shall give the dissection in the words of Mr. Murray.

"The face had a natural, composed appearance; and the rigidity of the body did not appear to be different from what is common. The right ear, and corresponding side of the face, as well as the scalp, exhibited a deep clay-blue colour. On the chest and belly, several spots and streaks, some green, others blue, were observed; and the back, upon which the body lay, was from head to foot of a livid colour; while several roundish spots, of a still deeper hue, gave to the shoulders and neck a mottled appearance. The penis was much swollen, and red. The scrotum also was enlarged, and of a dark blue colour.

"Upon opening the belly, the smell was not unusually offensive, and the abdominal contents did not appear to us to have undergone alteration after death; but, in the cavity, several ounces of a highcoloured liquid were found. On the intestines, jejunum and ilium, many purple spots, some of them several inches in circumference, were observed: and the outer surface of the stomach, in a tract which extended from the cardiac orifice, and occupied, for some distance downwards, the whole circumference of that viscus, excepting the small curvature, was of a clear, dark red colour; and through this space dark lines, apparently veins, ramified. This appearance, perhaps from 20 to 30 square inches in extent, was strongly marked in contrast with the natural state of the inferior extremity and small curvature. The substance connecting the stomach to the spleen, was, as well as a small part of the transverse colon, of a red colour. The spleen was gorged with blood; the liver healthy. The duodenum, from a small distance below the pylorus, almost to its inferior extremity, and round nearly the whole intestine, was of a very dark purple colour. Upon opening the stomach, the internal surface of that part where the outward appearance, already described, existed, was found of a bright colour, and over this lighter dots were thickly scattered; making such an appearance as might be produced by a red colour being dashed, from a painter's pencil, upon a somewhat darker *ground.* The inner coats of the duodenum were very dark coloured, with a slightly reddish hue, pulpy, thickened, and easily separated from the peritoneal covering, while in one roundish spot, of the size of a crown-piece, the villous and muscular coats were entirely wanting. Red patches were observed on the inner surface of the jejunum and ilium, the shape, size, and situation of which were the same as those of the appearances already noted on the outside of these intestines. The stomach and duodenum contained about a quart of a brown, semi-opaque, thickish liquid; the jejunum and ilium were empty, and coated with a yellow viscid matter. The lungs and heart were quite healthy; but in the cavity of the thorax were 10 ounces of a reddish turbid liquid, and about half that quantity in the pericardium. The pharynx was of an unusually red colour: the whole of the brain was healthy, and of firm consistence." *Edinb. Journ. p.* 171.

The symptoms in the three individuals who recovered, were, of course, milder considerably in degree, than in the case here detailed. All four had breakfasted together on the morning of their illness, on porridge consisting of milk, salt, and oatmeal. By each of the parties a dose of castor oil, and a course of weak Epsom salts had been taken. They had also entered on a medicine composed of alkaline solution and opium, but considering themselves beyond hope they left it off.

We grant, with Mr. Murray, that the *internal* evidence of poison here is very strong; but we do not think it conclusive, were there no *external* evidence of a more unequivocal nature. We put it to Mr. Murray whether, if no suspicion had existed in the minds of the parties poisoned, and this individual *alone* had applied to him, and been opened after death, he would have decided that he died of poison? The circumstances of four indiduals being taken ill at the same time, and with nearly similar symptoms, was a part of the *external* evidence, and has nothing to do with the internal or pathological evidence under consideration. The whole of the phenomena, both before and after death, were the product of *inflammation*, and although the man's confession puts it beyond a doubt, that arsenic was the *cause*, yet the same effects might and do ensue, from *other* causes; consequently, we do not see how the medical evidence could affirm, merely from the internal phenomena, living and post mortem, that the death was occasioned by arsenic. Nothing would induce us to give such an evidence, but the detection of the poison itself. It was for the jury to take external evidence to their aid, but we conceive the surgeon or physician had only the symptoms and dissection to guide them. We regret that Mr. Murray has not given the particulars of the trial, as it would form an interesting article in a forensic point of view. We hope he will publish the evidence.

---

25. *Voltaic Battery.** *It is known to the readers of this Journal, that Dr. Philip, some years ago, presented to the Royal Society, an account of some experiments, from which he inferred that, the functions of the stomach and lungs are interrupted by dividing the eighth pair of nerves in the neck, and restored by subjecting these organs to the influence of the voltaic battery; that these inferences were called in question by certain members of the Royal Society, who could not obtain the same results from Dr. Philip's experiments; that Dr. Philip, for the satisfaction of these gentlemen and the Royal Society, last year, repeated his experiments, with the assistance of one of the gentlemen in question, Mr. Brodie, at the Royal Institution, where the president and other members of the Royal Society, witnessed the results, and that Mr. Brodie, in the handsomest manner, then declared his conviction of the accuracy of Dr. Philip's statements.*

*The Royal Society, with that liberality and candour which are inseparable from a love of science, now publish the results of the investigation; which will do away the effects which naturally arose, from the report of the gentlemen abovementioned, having been received as a refutation of Dr. Philip's statement. That now published, is drawn up by Dr. Philip, in his own and Mr. Brodie's name. It is very short, but we have only room for the following extract.*

" In other experiments, in which, after the division of the nerves, the divided ends had been turned completely away from each other, little or no perfectly digested food, when the animal was allowed to live some hours, was found in the stomach, and the longer the ani-

---

* Some Positions respecting the Influence of the Voltaic Battery in obviating the Effects of the Division of the Eighth Pair of Nerves, drawn up by A. P. W. Philip, M.D. F.R.S.E. &c. and presented by B. C. Brodie, Esq. F.R.S.—(From the Philosophical Transactions of the present year.)

mal lived, the smaller was the proportion of the digested food found in the stomach; the great mass having the appearance of masticated food, which was not sensibly lessened in quantity, however long the animal lived. In an experiment in which, under such circumstances, the stomach was exposed from the time of the division of the nerves, to the influence of a voltaic battery, sent through the lower portion of the divided nerves, its contents were apparently as much changed, as they would have been in the same time in the healthy animal. The change was also of the same kind; the contents of the stomach assuming a dark colour, and those of the pyloric end being more uniform, and of a firmer consistence, than those of the central and cardiac portions of the stomach, while the whole contents became less in quantity."

---

26. *Surgical School of Edinburgh.** Mr. Liston is well-known as a junior surgeon of Edinburgh, who has performed and published, accounts of, some very perilous operations in surgery. He has passed the ligature for subclavian aneurism successfully, between the scaleni muscles—the fourth operation of the kind *only*, and almost the *only* successful one.—In another case of ossified aneurismal tumor, he removed the base of the scapula with success, &c. &c. &c. The Revue Medicale (September 1820, p. 117) observes of that latter operation, that it was "accompagnée de circonstances tres remarquables, et montre jusque quel peut la chirurgie peut etre entreprenante sans témérité". It is also well known that the simplicity of his methods of operating, their brevity and success, whatsoever the operations be, have been so conspicuous, that he must, at all events, point towards the summit of Scottish surgery, if not more. This is doing a great deal, but for this, it seems, he has encountered much of the same unrelenting persecution which deprived Edinburgh of the talents of Messrs. John and Charles Bell, and threw obstacles into the paths of a Brown and a Gregory. We have no inclination to enter into this inglorious conflict, but it is impossible not to feel indignant at the *uncontradicted* exposures in Mr. Liston's letters. Mr. L's cause we hope is too good to need our support, and his proposition of a new school of surgery deserves high encouragement, for we are declared enemies to *monopoly* in science, as well as in commerce. Nothing would so much exalt Edinburgh, as a school of surgery, founded on the same excellent system as that of medicine. It is to be feared, however, that Mr. L. will experience great obstacles to his scheme from the opposition of an unrelenting and arbitrary force, deafened by prejudice and based on personal influence and interest. Be this as it may, superior example will work: Dr. Duncan, jun. in his excellent Clinical

---

* Three letters to the Contributors to the Royal Infirmary, to the Lord Provost, and to the Managers of the Royal Infirmary, Edinburgh, by Robert Liston, Surgeon, &c. 1822.

Reports, candidly confesses that though the Edinburgh schools disavowed John Brown's doctrines, yet that their poison was so subtle and pervading that it decidedly influenced all their practice. It is to be hoped that somewhat of modern British feeling has had an alterative effect on former Northern habits; but Edinburgh has long been famed for these things, though most readers of those theories of Scotch wisdom and liberality in public economy, which appear in certain political journals, would look confidently for something better. We will venture to advise those luminaries who can see errors so acutely in our English institutions to reflect that there is some work done at home, which would not bear the liberal air in our generous country for an instant. This they not only know, but must opine, that the measures and principles which they most inconsistently advocate under their own domination, to sink genius at its first breath, are precisely those which, if pertaining to England, would call forth their bitterest animadversions.

In conclusion, we beg to state it as our belief, that Mr. Liston has been intemperate in his expressions, and too violent in his conduct—circumstances that may well admit of palliation, on account of his youth and zealous disposition; but no excuse can be offered for the rancorous abuse and vile vituperations launched forth by the hireling retainers of the law against a defenceless, youthful, and we will repeat, an excellent operating surgeon, in order to sacrifice his rising character, and *extinguish* his dawning merits at the shrine of a haughty oligarchy. But we confidently anticipate a salutary reaction against this ignoble persecution. Let the youth of England, Scotland, and Ireland, whose hearts are not yet rendered callous by self-interest and party cabal, do *justice*. Let them examine with their own eyes, and act with independence and generosity. We need not ask more, and we cannot expect less.

---

27. *Melanose.** This able and indefatigable anatomist and surgeon has found a black matter, which coloured linen and paper as much as Indian ink, in various parts and structures of the human body—frequently in the form of encysted tumours. On examination these degenerations presented no trace of vessels, nerves, or fibres. M. Breschet considers them as morbid secretions rather than decompositions of tissue. He has found them in various animals, as the dog, cat, hare, but particularly the horse. This black matter was sometimes fluid, pultaceous, hard, concrete, laminated. It is homogeneous, and without taste or flavour. It is miscible with water and alkohol.

The encysted melanoses vary in size from that of a pea to a pigeon's egg. When larger than this, M. Breschet thinks they are

* M. Breschet, considerations sur une alteration organique, &c. &c. &c. Journal de Physiologie, par M. Majendie.

conglomerations of several smaller ones. Our author has sometimes found these milanoses mixed with encysted tumours, containing a very yellow kind of fat, with mucilaginous and gelatinous substances.

Another form of these melanose productions is that of false membrane, or membranous expansions on the surfaces of the mucous and other tissues. Sometimes the black matter is extravasated, in the consistence of bouilli, into the cavities of the body. In some cancerous affections of the liver, intestines, and uterus, the serous infltrations are tinctured with this black matter.

This matter has been analyzed by Messrs. Barruel and Lassaigne, and these analyses lead to the conclusion that it is a deposition of the colouring matter of the blood, of fibrine, and of three distinct sorts of fatty matter. In many diseases we observe secretions of dark matters, as the black vomit in yellow fever, and black alvine excretions attending cancerous affections of the stomach and bowels. The black incrustations on the teeth, gums, and tongue, in low fevers, bear a strong resemblance, our author thinks, to the matter of melanoses, as well as the discharges in melena and hæmatemesis. M. Breschet is disposed to think that jaundice is produced by the blood rather than the bile—an opinion founded on necroscopical observations made in the Hospice des Enfans, on the bodies of jaundiced foundlings. The yellow suffusion which takes place soon after birth can hardly, he imagines, be produced by bile. He thinks it more natural to attribute this phenomenon to changes which take place in the circulation, the same as we observe after contusions, &c.

This is a very interesting and ingenious memoir, honorable to the author, and advantageous to the Journal in which it appears.

---

28. *Tetanus.** Tetanus, especially of the traumatic kind, is so dreadful a disease, and so very often fatal, that every successful case should have all possible publicity, for the good of humanity and the honour of the profession.† Mr. Barr's patient was a young man, who, in falling from a horse, was trampled on the belly by the animal. He went about his usual avocations, however, for seventeen days, when he was, all at once, seized with tremendous universal spasms, bending the head and trunk in the form of a bow—but whether forward or backward is not stated. Mr. B. saw him an hour after the attack, and the spasms were then recurring every five

---

* Mr. Geo. Barr, Surgeon, Kelsyth. Edinb. Journal, No. 71.

† Aretæus, with his usual terseness and forcible language, calls tetanus an "inhumana calamitas, injucundus aspectus, triste intuenti spectaculum, et *malum insanabile.*" Modern medicine, on this, as on very many other ocasions, can boast of much more success than the ancient. Tetanus cannot now be called "malum insanabile," even were it more fatal than it is.—*Ed.*

minutes. The neck was stiff and immoveable; and many of the muscles, especially the pectorals, felt rigid. The jaws were firmly locked. Our author instantly bled him, *pleno rivo*, to the amount of 50 ounces;* in about half an hour after which, the muscles of the jaw relaxed, and three fluid drachms of laudanum were exhibited. The spasms became less powerful and less frequent, having now an interval of half an hour. A drachm of laudanum was repeated after each spasm. At three o'clock next morning, the patient was again bled to 12 ounces; the laudanum, combined with two grains of camphor, to be continued as before. The spasms now returned about once in the hour, and not nearly so severe as before. Throughout the whole of the second day the paroxysms recurred once an hour, the jaws being completely locked during each paroxysm. Bled in the evening to 20 ounces. Having vomited the tincture, four grains of solid opium were ordered to be taken after each spasm. A nitric acid blister was applied to the whole spine. The spasms now occurred every two or three hours. On the third day some strong purgative pills, and three 15 grain doses of calomel were ordered to be taken. Had seven stools through the day, having taken the 45 grains of calomel. The spasms recurred this day every hour and half, and very severe. A drachm of solid opium was therefore given at once. In about 20 minutes the patient began to doze a little, but not to sleep—complained of giddiness, and some dyspnœa. In an hour and ten minutes he fell asleep, shortly after which, the breathing became slow and very laborious, the number of respirations being four in the minute. The patient having continued in this state about two hours, Mr. B. roused him, when he felt nausea, which was succeeded by full vomiting on taking some warm water. This produced much relief. *Fourth day.* No spasms since last night, except one paroxysm at 10 o'clock this forenoon. Mouth sore from the calomel. Venesection to 16 ounces, the pulse being 100 and full. *Fifth day.* No spasms—mouth very sore, and saliva flowing gently. From this time he became convalescent, and soon recovered completely.

We see in the above case that three powerful remedies were employed—venesection, opium, and mercury. We cannot therefore positively say which was the efficient medicine. Perhaps no one or two of them would have succeeded. The venesection prepared the way for the speedy operation of the opium and calomel. We have long been convinced that general and local (from the spine) bleeding, opium, and mercury, are the best means we possess of checking this formidable disease. These are the means indeed which have been employed of late years between the tropics, where tetanus is

* It appears from Celsus that Asclepiades was a great advocate for bleeding in tetanus. "Asclepiades utique mittendum sanguinem credidit." Celsus himself, among many other means, recommends cupping the spine, and then applying the actual cautery. We think this plan would probably be useful.—*Ed.*

so prevalent. The dose of opium, in the present instance, we think was rather too large, though we are quite satisfied that in tetanus and some other painful diseases, such is the torpor or insensibility of the stomach, that opium and all other medicines may be taken in doses *infinitely* greater than the same patients would be able to bear in a state of health. Purgatives are useful auxiliaries in recalling sensibility to the ganglionic system of viscera, and thus lessening the quantum of irritation on the origins of the spinal nerves. All the phenomena, indeed, of tetanus would lead us to conclude that nervous irritation of a high degree is propagated to the spinal marrow; and that, upon a well known and universal principle ("ubi irritatio ibi fluxus,") we may next expect the vascular system to be drawn, sooner or later, into a morbid state. The irritation, however, may be so great, and the spasms so violent, as to destroy life without leaving traces of vascular derangement on dissection. In many instances these vascular alterations have been detected; and whether they are detected or not, there can be no doubt of the nervous irritation in the first instance, and indeed throughout the whole of the disease,

---

29. *Fractures of the Clavicle.** Every surgeon knows the difficulty of keeping the fractured extremities of the clavicle in co-aptation. When this bone is broken, the internal portion is firmly fixed in its place by the costo-clavicular ligament, and by the opposite actions of the sterno-cleido and pectoralis muscles. It is therefore the external fragment which always becomes displaced. It is borne down by the weight of the arm and the action of the deltoid muscle, while it is, at the same time, drawn forward and inward by the pectoralis major, and thus carried under the internal portion, which forms an eminence over it. We all know that the figure of 8 bandage is very inadequate to the counteraction of the above circumstances. Desault invented an apparatus which appears to have been very successful in his hands, but not so in those of others after him. The plan of Mr. Charles Bell, by means of the double-headed roller and compress under the arm, seems better adapted to general application than that of Desault or Boyer.

"But, says Dr. Brown, as the force exerted to keep the external portion raised in contact with the internal, depends upon the short turns around the shoulder, a very slight stretching of the bandage will occasion it to be displaced; and unless the bandage be frequently re-applied, it cannot be retained in its situation.

"Take a single-headed roller, eleven yards long and three and a half inches broad,† and place one end of it a little forward of the

---

* A new mode of bandaging fractures of the clavicle. By Stephen Brown, M.D. of New York. Amer. Med. Recorder, No. XVI.

† A bandage narrower than this will, of course, be found more convenient in cases of children axilla of the opposite side; carry the roller from thence across the upper part of the chest under the armpit of the affected side, and around the body to meet the end, over which let it lap a little, and pin or sew it fast. Place a cushion in the armpit, as directed by Desault, which is to be attached to the bandage thus passed around the body by tapes, or, which is better, let it be sewed. The cushion being fixed, the surgeon seizes the patient's elbow, the forearm being bent to a little less than a right angle with the arm, and brings it forward, upward, and inward, pressing it closely against the body. Let the shoulder be sufficiently raised, so that the external fragment be carried up to its place, and the deformity entirely disappear. Let an assistant hold the elbow and forearm in this situation, while the surgeon brings the bandage down obliquely across the breast over the forearm nearly across its middle, passing it around under the elbow, and across the back obliquely upward to the lower part of the scapula of the opposite side. Here it should be pinned or sewed to that part which was first passed around the body; then with a turn carry it over the shoulder of the same side, and down obliquely across the breast, as before, overlapping the first cast about two-thirds, and across the forearm, nearer the elbow than the first; carrying it around under the elbow, and up across the back, to be fastened upon the preceding cast in the same situation where that was attached to the first. With a turn carry it over the shoulder, where it should nearly cover the preceding cast; then down across the breast as before, overlapping the preceding fold as the second does the first. Four or five of these casts over the forearm and elbow are sufficient. Let the last two or three embrace and support the elbow, with such a degree of force as to keep the shoulder well raised. Fasten the cast which was last carried around to as many folds of the preceding ones as it will cover, just forward of the elbow, upon the forearm;—then with a turn carry it over the arm just above the elbow, across the back, under the axilla of the opposite side, and around and across the chest, to be attached to the folds upon the forearm, as before. Then with a turn overlap the first cast, and carry it around in the same manner.

"Two or three of these casts are sufficient; the object of which is to keep the lower extremity of the humerus closely in contact with the body, which, by the aid of the cushion, keeps the shoulder outward.* The hand may be supported in a sling formed of the last extremity of the bandage, the last cast of which may be pinned

* In females in order to obviate, as much as possible, any inconvenience by pressure upon the mammæ, the casts which are carried over the humerus, when they are brought under the armpit of the opposite side, instead of being continued directly across the breast to the lower part of the humerus of the affected side, may be carried obliquely upward toward the neck, upon the casts that were passed over the shoulder; to which let them be fastened. Then with a half turn continue down upon the forearm near the elbow, and fasten again; then with a turn carry it over the lower part of the humerus, &c. &c.

to the folds upon the back, and the end brought over the shoulder to the wrist; or, attached to the folds upon the breast, and the end passed around the wrist and hand once or twice, and fastened again.

"Before applying the bandage, a cushion of several folds of soft linen should be laid upon the shoulder to prevent irritation, over which the folds of the bandage should rest. To answer the same purpose, the arm and forearm may be covered with a layer of soft linen, and a few folds of the same placed in the armpit of the opposite side.

"The bandage being applied, let the casts, where they overlap, be sewed to each other in every situation where the bandage is most likely to be deranged. This is particularly necessary where they cover the arm and forearm.

"This mode of bandaging will be found to possess equal facilities with the most approved plan of reducing fractures of the lower extremities."—*Medical Recorder, No.* XVI. *p.* 657.

---

30. *Protecting and Modifying Influence of Vaccination.** The *unqualified* protection of the vaccine process against variola, is now properly given up by all unbiassed practitioners. It is far better for either a man or a medicine, that his or its character and value should be precisely estimated and ascertained, than that either should be exaggerated above, or depreciated below, the standard of truth. Vaccination, like all other things in this world, must submit to the imputation of imperfection. Fortunately for itself, and for mankind, it can admit this draw-back, and still hold up its head with honour. It may be said of vaccination that, in most instances, it stems variola entirely—and in all, or nearly all instances where it fails in preventing, it so modifies the variolous disease as to leave it scarcely cognizable as the same affection. Is not this enough?

Among the now innumerable proofs of the above position, may be found some cases and facts brought forward by Dr. James Reed in our respected cotemporary of Edinburgh, for April last. A stranger with variola from Glasgow fell ill at Kilmarlock, and died on the 13th day. The contagion issued from this focus, and its steps were equally well marked and distinctly traced. Its first attack was on a vaccinated girl. "After a smart fever, the vesicular eruption did make its appearance; and moderate chicken pox, ending safely about the eighth day of eruption, was the consequence." Precisely opposite to the house where the stranger died, a mother and her infant at the breast (both unprotected) caught the contagion. In the mother, severe and confluent small-pox terminated fatally on the 10th day of the eruption. The infant escaped, but bears the permanent marks of variola. The husband of this woman (himself

---

* Dr. James Reed, of Kilmarlock. Edinb. Journal, No. 71.

inoculated) had assisted the stranger above alluded to, and thus carried home the contagion to his family, though it made no impression on himself. A little farther on, in the same side of the street, two vaccinated children took the disease. The eruption answered precisely to the description of "modified small pox." The recovery of these infants was rapid. Curiosity (foolish curiosity) led a young man, who had been early vaccinated, from another village a mile distant, to visit the stranger who died. He caught the modified pox, which terminated safely in six days. In another part of the town a woman, inoculated in infancy, and bearing slight marks of small-pox in different parts of the body, had a vesicular eruption, (after a pretty severe attack of fever) at first on her face and temples, then on the arms, hands, lower limbs, and feet, but very partially on the body. The eruption on the face dried up about the fourth day, and soon scaled off. That on the extremities advanced slowly to maturation, increased in size, coalesced, and formed large and deep pustules, some of which were not dried up on the 12th day. This woman had inadvertently called at the house of the last-mentioned patient, and sat chatting with him for some time. She had two children, both vaccinated. Neither of them suffered from the mother's disease.

The above are the main facts of Dr. Reed's communication, and the inference to be drawn from them is too obvious to require a word. They go to the corroboration of the position laid down at the commencement of this notice. We agree with Dr. Reed that it is highly gratifying to think what a vast proportion, in the late epidemics, resisted the utmost exposure to the influence of variolous contagion. For one case in which vaccination failed in any measure to secure the constitution, ten have shown themselves invulnerable, even where the mischief seemed most concentrated. As for death from variola after vaccination, it is so rare as scarcely to be worth mentioning. Dr. Reed relates an instance of the *speedy* mode in which vaccination extends its protecting influence. A young man from Kilmarlock was going through the wards of the Royal Infirmary at Glasgow, in one of the side rooms of which, a child lay covered with small-pox. The youth, who had never been vaccinated, felt an involuntary shuddering at the sight of the disease; and in a day or two after his return from Glasgow, fell ill, and small-pox eruption came out in due time. At this moment it was ascertained that his nurse had never had the small-pox, or went through any protecting process. She was immediately vaccinated in both arms. The operation succeeded well; and although she was about the patient constantly during several weeks, in a situation peculiarly unpleasant, she escaped with perfect impunity. Finally, Dr. Reed expresses his conviction that small-pox, the varioloid disease, and varicella, are only different modifications of one and the same disease.

